# Post-burn breast reconstruction using an artificial dermis—a long-term follow-up

**DOI:** 10.1186/s41038-016-0037-9

**Published:** 2016-07-04

**Authors:** Yoav Gronovich, Adi Maisel Lotan, Meir Retchkiman

**Affiliations:** Department of Plastic and Reconstructive Surgery, Shaare-Zedek Medical Center, 12 Beith Street, Jerusalem, Israel

**Keywords:** Burned breast, Integra, Artificial skin, Burn reconstruction

## Abstract

**Background:**

Full thickness burns of the chest in childhood are a devastating problem that requires challenging reconstructive options.

Integra is a bilaminate artificial dermis composed of shark chondroitin 6-sulfate and bovine collagen. The dermal matrix serves as a scaffold for fibroblasts and endothelial cells. Vascularization of the matrix begins after 2–3 weeks, and eventually, the matrix incorporates with the tissue to create a new dermis. The main advantage of the Integra is that the neodermis is of the same quality as a native dermis.

**Case presentation:**

In this case report, we present post-burn breast reconstruction of a 12-year-old girl using Integra, with a long follow-up of 7 years. To the best of our knowledge, there is no published follow-up of breast development after reconstruction with Integra from its beginning point at the age of puberty until after the growing process has terminated.

**Conclusions:**

Integra is a reliable reconstructive tool for burned breast. If done before puberty, it can help in getting normal developing tissue with satisfying esthetic results of size, shape and symmetry.

## Background

Full thickness burns of the chest in childhood are a devastating problem, because of the soft tissue damage that prevents the breast from developing in puberty. The scar tissue of the breast causes contractures that result in size, shape, and positioning problems [1]. The reconstructive options are challenging because none of them can evade the scar tissue and each of the alternatives has its drawback. The most frequent reconstruction of the burned breast includes excision of the contracted tissue and a split thickness skin graft above it. Other reconstructive modalities include full thickness skin graft, local fasciocutaneous, or musculocutaneous flaps or the use of an artificial dermis [2, 3]. Integra (LifeSciences Corp., Plainsboro, NJ, USA) is a bilaminate artificial dermis composed of shark chondroitin 6-sulfate and bovine collagen. The dermal matrix serves as a scaffold for fibroblasts and endothelial cells. Vascularization of the matrix begins after 2–3 weeks, and eventually, the matrix incorporates with the tissue to create a new dermis. The main advantage of the Integra is that the neodermis is of the same quality as a native dermis [4–7]. Studies of reconstruction with Integra showed promising results [1–6, 8, 9].

In this case report, we present post-burn breast reconstruction of a 12-year-old girl using Integra, with a long follow-up of 7 years. To the best of our knowledge, there is no published follow-up of breast development after reconstruction with Integra from its beginning point at the age of puberty until after the growing process has terminated.

## Case presentation

**Fig. 1:**
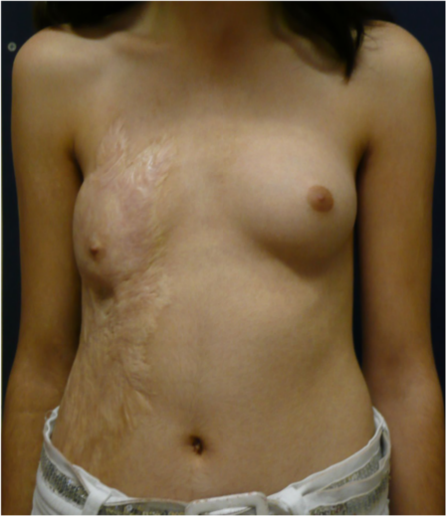
Burn of right breast—12 years old

**Fig. 2:**
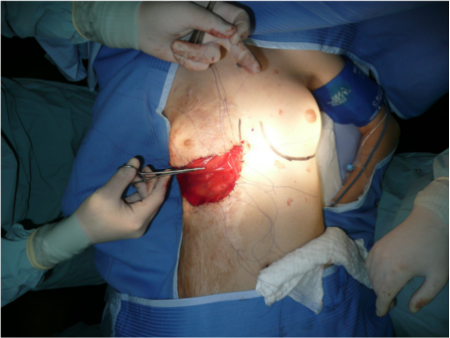
At the operation after releasing the contracture and putting the Integra on the breast (first operation)

**Fig. 3:**
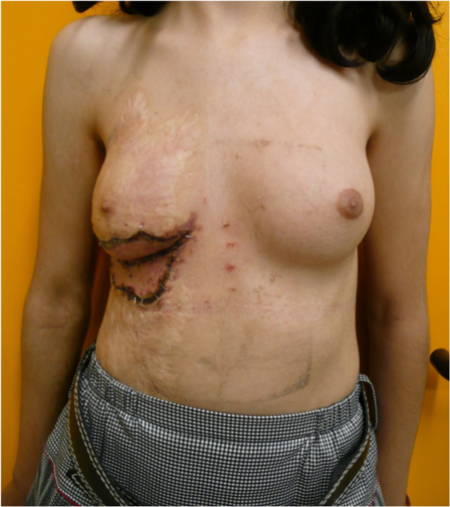
After putting the skin graft above the Integra (second operation)

**Fig. 4:**
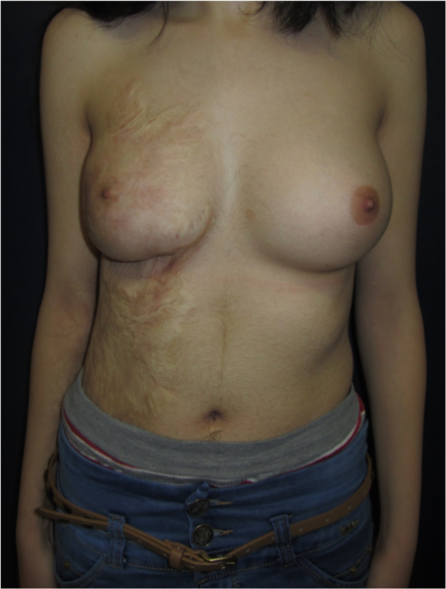
Follow-up—19 years old

A 12-year-old healthy girl had suffered a deep third degree scald burn at the age of 2 years. The burned area covered all of her right chest wall area. It was treated conservatively with no skin grafting. At the beginning of puberty, the right breast tissue was unable to grow because of the contracted lower pole (Fig. 1). In the preoperative planning, an Integra 10 cm × 15 cm was selected. The patient was operated under general anesthesia. First, the contracted area was excised and a gap of 7 cm× 5 cm resulted. The unmeshed Integra was putted above the exposed area and attached to the skin borders with absorbable sutures (Vicryl 4/0) (Fig. 2). A tie-over dressing with Xeroform and sponge was putted above the Integra and was stapled to the skin. Operative and postoperative management were uneventful. After 4 weeks, the patient went through the second operation. Under general anesthesia, the silicone layer of the Integra was removed and a thin split skin graft (0.01 in.) taken from the anterior aspect of her thigh was put on the integra and attached to it with absorbable sutures (Vicryl 4/0) and staples. A tie-over dressing with Xeroform and sponge was putted above it. The operative and postoperative management were uneventful. After 4 days, the skin was taken and the dressing was exchanged (Fig. 3). Follow-up of the patient included measurements of the suprasternal notch to nipple distance (SN-N), nipple to inframammary fold distance (N-IMF) and graft size (Table 1). The patient was seen until the age of 19 (7 years post operation) with satisfying results and symmetric development of her breasts (Fig. 4).

**Table 1:** Measurements of the developing right and left breast

		SN-N (cm)		N-IMF (cm)		Graft size (cm)
		Right breast	Left breast	Right breast	Left breast	
Age (years)	12	17.5	14.5	2.5	5.5	7.0 × 5.0
	13	16	15	4.5	5.5	7.2 × 5.2
	14	15	15	5.5	5.8	7.4 × 5.3
	15	15.5	15.5	5.7	6	7.5 × 5.7
	16	16	16	6.5	6.5	7.7 × 6.0
	17	17	17	7	7	7.7 × 6.5
	18	17	17	7	7	7.7 × 6.5
	19	17	17	7	7	7.7 × 6.5

*SN-N* suprasternal notch to nipple

*N-IMF* nipple to inframammary distance

### Discussion

Reconstruction of the post-burn breast includes excision of the contracted scar, releasing of fibrotic tissue and covering it with one of the following modalities: split thickness skin graft, expanded full thickness skin graft, musculocutaneous or fasciocutaneous flaps, or an artificial dermal matrix.

Split thickness skin grafting is very common due to its ease of harvest and availability. It can be meshed up to 1:9, thus enabling a large area of coverage. Yet, it assumed to cause contraction of the reconstructed area because of secondary contraction forces of the graft. This drawback should be considered when one chooses this modality for areas of esthetic and functional importance as in our case.

The full thickness skin graft is preferable because of better esthetic results and less secondary contracture of the skin. Its main drawback results from the necessity to first expand the donor site in order to get enough skin for reconstruction. This means another operation with a period of a few months of inconvenience until the expansion phase is completed. Moreover, consideration should be taken of the risks and possible complications regarding the expanders (extrusion, infection, damage to the skin, etc.). In our case, the patient was 12 years old when she first came to us. The use of an expander in her abdomen was assumed to be hard to tolerate.

Musculocutaneous or fasciocutaneous flaps are other alternatives for reconstruction, with good ability of coverage and which can be matched to the defective area, thus leading to very good functional and esthetic results. The main drawbacks of these alternatives are donor site morbidity and the risks of complications and failure.

The artificial dermis has become more popular in recent years for reconstruction because of its simplicity and the predictability of the reconstructive results. The biodegradable dermal replacement layer serves as a matrix for the macrophages, fibroblasts, and endothelial cells from the host tissue. It causes a new dermis to grow, thereby creating a functional tissue instead of the excised one. The only donor site that is needed is a very thin split skin graft for final closure after the dermal graft has taken. The main drawback of this reconstructive modality is its price which is very expensive.

In the patient herein presented, we decided to use an artificial dermis matrix to reconstruct the defect. This method has been described in the literature for variety of reconstructions including burned breast [1, 3]. Palao et al. used artificial dermis in reconstruction of burned breast of 12 patients. They showed in their study that after 1 year, the host collagen completely replaces the artificial one, and elastic fibers were observed in the dermal regeneration template. They had high satisfaction rate after 1 year of follow-up (1). Two of their patients were at puberty age during the reconstruction (13 and 14 years old), though, they had no follow-up for more than 1 year, so there is no way to get knowledge from their study of the long-term development of the reconstructed breast.

Our case is the first to be published that use this method for breast at the beginning of the puberty and has follow-up of 7 years until the end of the puberty period at 19 years old. By that, we showed that the burned breast developed as well as the other breast, with satisfying symmetric size and shape. The importance of this case is that it emphasizes the long-term advantage of Integra as a reconstructive tool for having a natural functional tissue.

## Conclusions

Integra is a reliable reconstructive tool for burned breast. If done before puberty, it can help in getting normal developing tissue with satisfying esthetic results of size, shape, and symmetry.

## Consent statement

Written informed consent was obtained from the patient for publication of this case report and any accompanying images. A copy of the written consent is available for review by the editor-in-chief of this journal.

## References

[1] Palao R, Gomez P, Huguet P (2003). Burned breast reconstructive surgery with Integra dermal regeneration template. Br J Plast Surg.

[2] Driver VR, Lavery LA, Reyzelman AM (2015). A clinical trial of Integra template for diabetic foot ulcer treatment. Wound Repair Regen.

[3] Tsoutsos D, Stratigos A, Gravvanis A (2007). Burned breast reconstruction by expanded artificial dermal substitute. J Burn Care Res.

[4] Leffler M, Horch RE, Dragu A (2010). The use of the artificial dermis (Integra) in combination with vacuum assisted closure for reconstruction of an extensive burn scar—a case report. J Plast Reconstr Aesthet Surg.

[5] Moiemen N, Yarrow J, Hodgson E (2010). Long term clinical and histological analysis of Integra dermal regeneration template use and literature review of template histology. Plast Reconstr Surg.

[6] Lee LF, Porch JV, Spenler CW (2008). Integra in lower extremity reconstruction after burn injury. Plast Reconstr Surg.

[7] De Angelis B, Gentile P, Tati E (2015). One-stage reconstruction of scalp after full-thickness oncologic defects using a dermal regeneration template (Integra). Biomed Res Int.

[8] Lee SM, Stewart CL, Miller CJ (2015). The histopathologic features of Integra dermal regeneration template. J Cutan Pathol.

[9] Verbelen J, Hoeksema H, Pirayesh A, et al. Exposed tibial bone after burns: flap reconstruction versus dermal substitute. Burns. 2015. doi:10.1016. 10.1016/j.burns.2015.08.013 26376411

